# Patient Health Record Protection Beyond the Health Insurance Portability and Accountability Act: Mixed Methods Study

**DOI:** 10.2196/59674

**Published:** 2024-11-06

**Authors:** Hemang Subramanian, Arijit Sengupta, Yilin Xu

**Affiliations:** 1 Florida International University Miami, FL United States

**Keywords:** security, privacy, security breach, breach report, health care, health care infrastructure, regulatory, law enforcement, Omnibus Rule, qualitative analysis, AI-generated data, artificial intelligence, difference-in-differences, best practice, data privacy, safe practice

## Abstract

**Background:**

The security and privacy of health care information are crucial for maintaining the societal value of health care as a public good. However, governance over electronic health care data has proven inefficient, despite robust enforcement efforts. Both federal (HIPAA [Health Insurance Portability and Accountability Act]) and state regulations, along with the ombudsman rule, have not effectively reduced the frequency or impact of data breaches in the US health care system. While legal frameworks have bolstered data security, recent years have seen a concerning increase in breach incidents. This paper investigates common breach types and proposes best practices derived from the data as potential solutions.

**Objective:**

The primary aim of this study is to analyze health care and hospital breach data, comparing it against HIPAA compliance levels across states (spatial analysis) and the impact of the Omnibus Rule over time (temporal analysis). The goal is to establish guidelines for best practices in handling sensitive information within hospitals and clinical environments.

**Methods:**

The study used data from the Department of Health and Human Services on reported breaches, assessing the severity and impact of each breach type. We then analyzed secondary data to examine whether HIPAA’s storage and retention rule amendments have influenced security and privacy incidents across all 50 states. Finally, we conducted a qualitative analysis of textual data from vulnerability and breach reports to identify actionable best practices for health care settings.

**Results:**

Our findings indicate that hacking or IT incidents have the most significant impact on the number of individuals affected, highlighting this as a primary breach category. The overall difference-in-differences trend reveals no significant reduction in breach rates (*P*=.50), despite state-level regulations exceeding HIPAA requirements and the introduction of the ombudsman rule. This persistence in breach trends implies that even strengthened protections and additional guidelines have not effectively curbed the rising number of affected individuals. Through qualitative analysis, we identified 15 unique values and associated best practices from industry standards.

**Conclusions:**

Combining quantitative and qualitative insights, we propose the “SecureSphere framework” to enhance data security in health care institutions. This framework presents key security values structured in concentric circles: core values at the center and peripheral values around them. The core values include employee management, policy, procedures, and IT management. Peripheral values encompass the remaining security attributes that support these core elements. This structured approach provides a comprehensive security strategy for protecting patient health information and is designed to help health care organizations develop sustainable practices for data security.

## Introduction

### Overview

The HIPAA (Health Insurance Portability and Accountability Act) [1] was enacted by the United States Congress aiming to develop a set of guidelines by which personally identifiable information maintained by health care providers and insurers is protected from fraud, theft, and unauthorized access. At the most general level, HIPAA prevents health care providers from disclosing such information to anyone other than the patient herself and their health providers without explicit consent from the patient. Since its enactment in 1996, it has been implemented in all states of the United States and is widely accepted by both patients and medical providers [2]. HIPAA is now a globally accepted public health law that governs the protection and security of patient health information. Many countries globally also implement various aspects of HIPAA compliance as a measure of security and protection of personal health record (PHR) data [3].

Presently, HIPAA is enforced, and failures to adhere to the act may result in civil and criminal penalties. While the enactment of HIPAA has certainly increased the privacy of patient information, the attempts at subverting the protection and illegally accessing private information have also increased multiple fold. In this paper, we analyze a publicly available dataset related to data breaches in health care systems with both quantitative and qualitative methods to decide how effective HIPAA has been and what current health care systems may need to do to protect individuals from such breaches.

In HIPAA, Privacy and Security Rules work together to protect the privacy and security of individuals’ PHR information. The Privacy Rule focuses on limiting how PHR can be used and disclosed, while the Security Rule focuses on protecting the confidentiality, integrity, and availability of electronic protected health information through technical and administrative safeguards. Both rules are enforced by the Department of Health and Human Services (HHS) and aim to protect individuals’ health information from unauthorized access, use, and disclosure. Table 1 compares the HIPAA Privacy Rule with the HIPAA Security Rule with respect to the most common attributes that concern health care data protection.

**Table 1:** Comparing the HIPAA (Health Insurance Portability and Accountability Act) Privacy Rule and HIPAA Security Rule across various features of the rule.

Features	HIPAA Privacy Rule	HIPAA Security Rule
Purpose	Protects the privacy of an individual’s health information	Protects the security of ePHI^a^
Covered entities	Health plans, health care clearinghouses, and health care providers	Covered entities and their business associates
Privacy standards	Limits how covered entities can use and disclose PHI^b^	Ensures confidentiality, integrity, and availability of ePHI
Required measures	Covered entities must have policies and procedures in place to protect PHI	Covered entities must implement administrative, physical, and technical safeguards to protect ePHI
Access controls	Covered entities must limit access to PHI to authorized individuals	Covered entities must implement technical policies and procedures to control access to ePHI
Data encryption	Required only for ePHI transmitted over an open network	Required for all ePHI, both in transit and for storage of data
Breach notification	Covered entities must report breaches of PHI to affected individuals and the HHS^c^	Covered entities must notify affected individuals, HHS, and the media (for large breaches) of any breach of unsecured ePHI
Enforcement	Enforced by the HHS OCR^d^	Enforced by the HHS OCR and the HHS Office of the Inspector General

^a^ePHI: electronic protected health information.

^b^PHI: protected health information.

^c^HHS: Department of Health and Human Services.

^d^OCR: Office for Civil Rights.

Despite stringent HIPAA rules affecting both the privacy and security of PHR data and their implementation, we observe an increase in attack surface and loss of PHR across US hospital systems. In this paper, using health vulnerability data from hospital systems over 15 years, we study the following research question: What are the best practices to protect the security and privacy of PHR data in hospital information systems in addition to HIPAA, state-specific security guidelines, and Omnibus regulations?

To answer the above research question, we use an extensive dataset of health data breaches from January 2009 to January 2023 published by the US HHS [4]. First, we analyze different health data system vulnerabilities by finding the correlation between the number of users affected and the various parameters and types of vulnerabilities. We further look at the different types of vulnerabilities, create clusters of states based on the extent of HIPAA adoption, and analyze vulnerabilities between these clusters. We then introduce the Omnibus Rule as an exogenous shock to see the effect of this rule on the breaches. Finally, we create a cluster of the types of vulnerabilities based on a qualitative analysis of the breach information description to identify the security “values” that are important to protect health care systems, which should provide guidance to future protection systems.

In summary, the aim of this paper is to analyze the data from existing health care security breaches and cross-compare the information against the extent of HIPAA adoption across the states (spatial) as well as the effect of the Omnibus Rule (temporal) to provide key health care security guidelines.

### Background and Literature Review

Much of the health care privacy and security literature falls in the realm of PHRs, specifically electronic health records (EHRs). Unlike other security-intensive information such as banking and taxation, health records have more transparency and must be viewed by and shared with multiple health personnel. Therefore, privacy and security are important drivers in adopting health data, particularly PHRs. A 2008 Markle Foundation survey [5] of 1580 adults reported privacy concerns related to the misuse of personal data by marketers (77%), employers (56%), and insurers (53%). While the report states that American people overwhelmingly believe that EHRs can improve their health, most participants were concerned about privacy factors. The main areas of concern were identity theft and limitation of employment opportunities due to specific health conditions and hence preferred to control who could access their health information.

Other studies also concur with the above findings. A study by Keselman et al [6] specifically mentions that security in PHR access must be configured to support privacy and security without violating HIPAA requirements. In another focus group study by Kerns et al [7], participants strongly opposed commercially developed PHRs and cited issues with privacy, security, and accuracy as a deterrent to adoption. Several studies identify potential security concerns related to data sharing, tampering, improper use, and illegal tracking as well as manipulation by third parties [8,9]. Despite the security concerns, new patients mostly prefer to engage electronically for their PHRs [6,7] so enhancing the security of the systems and databases that are used for the storage and transmission of such data has become a priority.

Despite the potential concerns among users about having highly personal health information in shared electronic infrastructure, having massive amounts of information of health information allows health providers to better identify and manage high-risk and high-cost patients, thereby benefiting both the health providers as well as patients [10]. This has been made using clinical analytics—big data techniques to reduce the costs of health care by identifying high-cost patients, readmissions, triage, decompensation, adverse events, and treatment optimization for diseases affecting multiple organ systems. Such techniques require careful aggregation and data integrity techniques to ensure that all associations are privacy-preserving and not subject to inappropriate tampering [11].

While the HIPAA regulations do provide the possibility of comparative effectiveness studies, they also introduce barriers including inconsistent institutional review board policies and complicated and costly procedures to obtain the consent of patients for release of their information. This necessitates the development of a new policy framework that will allow and encourage the use of health information in all forms—fully identifiable, partially anonymized, and deidentified [12].

Development of a new framework around providing security to health information will require the reduction in ambiguity and confusion surrounding the concept of “health security,” particularly when combining information from diverse global sources. To counteract this, the global public health community will need to develop a common understanding of the security protocols that involve stakeholders in developing countries, industrialized countries, the humanitarian community, as well as military organizations [13].

Multiple techniques have been proposed in the literature for securing and preserving the anonymity and privacy of EHR clients’ symmetric and asymmetric keys for deidentification [14]. Access control, encryption, and auditing tools have also been proposed and developed [15]. Health care organizations are also developing privacy architecture using a combination of enterprise data warehouse and a software intelligence and analytics layer to counteract these threats. One such system developed by Houston Methodist Hospital [16] uses methods like specialized derecognizable proof of information, limited information access, and security controls in the hidden specialized stages to safeguard patient protection. Many other current efforts also use different forms of anonymization and encryption to preserve security and privacy [17]. The most prominent other features that are essential in such privacy preservation include system and application access control, compliance with security requirements, interoperability, integration and sharing, consent and choice mechanisms, policies and regulation, applicability and scalability, and cryptography techniques [18].

Prior research by Peddicord et al [12] similarly identifies barriers to research initiatives imposed by HIPAA and provides a policy-based framework as a collaborative mechanism for creating a process of managed consent and development of a research safe harbor to protect the privacy of health information. Similar governance frameworks have also been reviewed in general public and government sector implementations [19]. Having partnerships between the public and private sectors in such governance is important to ensure such reforms are successful in any information and communication technology solutions [20].

Other comparable techniques used in the literature for maintaining integrity in big data in EHR include blockchain-based solutions [11,21]. Privacy issues surrounding health data have become increasingly prevalent in recent years, particularly with the rise of EHRs and other digital health technologies.

The sensitive nature of health data makes it particularly vulnerable to privacy breaches, which can have serious consequences for individuals, health care providers, and health care systems. Recent studies have found that blockchain-based EHRs can give patients greater control over their data while ensuring their information is kept secure and confidential. Similarly, blockchains can be used to create a secure and private platform for sharing medical data among health care providers without compromising patient privacy [19].

### HIPAA Privacy Rule and Security Rule

To protect health data across multiple sources and stakeholders, various safeguards have been researched in the literature. These methods include physical safeguards such as physical locks and other access control methods for computers and other equipment; technical safeguards such as encryption, firewalls, strong passwords, antivirus and antimalware systems, mobile agents, and dual authentication schemes; and administrative safeguards such as risk assessments, personal device use policies, and deidentification of samples [22].

Finally, there is literature on the agreement of multiple states or stakeholders toward the development of a collaboration agreement, and harmonizing the state privacy laws and measures would be a vital component of the implementation of these [23]—our work does provide an analytical view of how the implementation of HIPAA across state barriers makes a difference in the effect of data breaches, and our analysis suggests that states with higher protection level than HIPAA are typically far better in terms of the effect any data breaches may have.

### Creation of the Omnibus HIPAA Rule

On January 17, 2013, the HHS released the long-awaited “Omnibus Rule.” The Omnibus Rule implemented most of the privacy and security provisions of the Health Information Technology for Economic and Clinical Health Act. The rule made the following changes to the security and privacy of health data providers and health systems: (1) extended the reach and limits of HIPAA with respect to obligations to business associates and their subcontractors, (2) modified the breach notification standard and expanded patient rights to access and restrict disclosure of protected health information (PHI), (3) imposed new rules governing the uses and disclosures of PHI, (4) clarified enforcement approaches, and (5) addressed obligations under the Genetic Information Nondiscrimination Act of 2008.

Of these changes implemented by the HHS, rules 1-4 impact the security and privacy of user data stored on health care information systems and are expected to significantly improve it. This significant change enabled several covered entities (such as hospitals, partners, and clinics) to analyze gaps in their security practice and perform the following services: (1) revising privacy and security policies and procedures; (2) revising breach notification policies, procedures, and breach response plans; (3) amending notices of privacy practices (and making sure the revised notices are properly posted and distributed); (4) training the workforce and promoting more ongoing awareness about privacy and security policies; (5) revising business associate contract templates and process of amending or renegotiating each one within the framework of the Omnibus Rule; (6) determining whether any forms, such as requests for access, should be updated or created; (7) continuing—or making an increased effort—to take advantage of the safe harbor provision by encrypting PHI according to HHS’ guidance; and (8) making sure an updated risk analysis is in place and reflects vulnerabilities addressed in HHS guidance, such as mobile devices.

## Methods

### Ethical Considerations

Ethics approval is not required for this study, as human participants were not involved.

### Study Design

First, we used data from HHS breaches and analyzed the severity of breach types and the impact of those breaches. Second, using secondary data and publicly available information, we tried to analyze whether data storage and retention changes to rules of HIPAA played any role in reducing security and privacy violations across the 50 US states. Finally, we used the textual data present in the vulnerability or breach data to analyze and recommend best practices for health care clinics and hospitals by doing a detailed qualitative analysis. We believe that hospital systems can alleviate the major concerns facing their health care systems overall.

**Figure 1:**
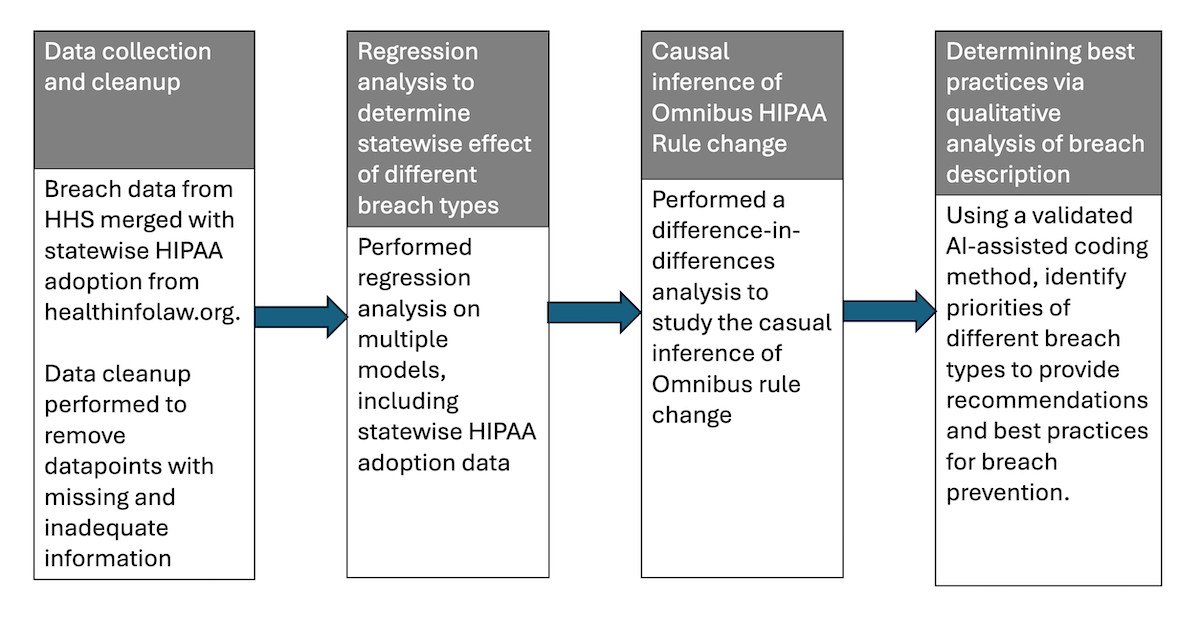
Research design flow. AI: artificial intelligence; HHS: Department of Health and Human Services; HIPAA: Health Insurance Portability and Accountability Act.

The primary dataset used in this study was HHS breach report data from 2009 until January 2023 [4]. Using this dataset, the different types of data breaches were analyzed by analyzing their statistics. We further developed a model that studies the extent of the effect of a particular type of data breach and the size of such an effect on the user. We supplemented these data with data about 50 US states from the Report on Adoption of HIPAA from HealthInfoLaw.org. We analyzed 2 major events in our sample. First, we used a difference-in-differences analysis to examine the presence of state-specific health data storage rules that are more stringent than the current HIPAA regulations, in order to determine how states with only HIPAA compared to those with stricter rules. Second, we analyzed the impact of the Omnibus Rule change, in relation to state-level HIPAA stringency, on the overall number of customers. Further, based on our analysis of breach descriptions, we determined the severity of breach categories to determine the key security “values” that guide future security practices. The sequence of analyses performed in this paper is summarized in the research design flow diagram of Figure 1.

The final step was to perform a ground-up qualitative analysis of the breach descriptions provided in the dataset to classify distinct topics within the dataset. Each code in this analysis represents a portion of data that are tagged as relevant to one of several predetermined categories. Each category represents a specific aspect of data breach reports that helps organize the data to make it easier to understand patterns, correlations, and trends. The resulting categorization of the codes thus represents research themes that determine the key security “measures” that can lead to best practices for identifying and preventing future breaches.

With over 3000 data points in the dataset, we faced a potential research dilemma—performing a qualitative analysis on such a large dataset could potentially be time- and resource-intensive. To automate this process, we used ATLAS.ti, a well-known research coding tool that uses machine learning algorithms to analyze qualitative data and identify patterns and themes. Based on the information provided by ATLAS.ti, the system internally used ChatGPT 3.5 developed by OpenAI as the model to conduct fully automated inductive coding. The motivation to apply the technique instead of manual coding is that this process of semiautomated coding can save time and potentially improve the consistency of the codes since the dataset is too large to code manually. However, to ensure that the codes generated by the system are on par with human coding, one of the researchers manually coded a small subset (n=50) of the breach descriptions using NVivo (version 14; Lumivero), followed by performing the automated coding on the same dataset by ATLAS.ti. An interrater reliability analysis was performed on the resulting codes. The Cohen κ score for this analysis was 0.88, indicating a reliable coding performance from ATLAS.ti. We posited, then, that using ATLAS.ti for the qualitative analysis would be sufficient for the entire dataset. A complete comparison analysis is included in Multimedia Appendix 1.

To be more specific, the codes were created by ATLAS.ti using large language models from the GPT family with the foundation of vast amounts of different texts and additional training by the researchers. During the procedure, the system split the text from the dataset into smaller chunks and sent these data to the GPT models for repetition analysis. The results were then combined algorithmically to generate an optimal mix of codes including various topics while avoiding too many redundant codes.

Serving as the application of large language models, GPT is sensitive to the surrounding text as the context to have a window of attention that ensures that the size of the data does not overflow the model. Thus, the contexts generated are not greater than 100 characters long and are broken up at natural paragraph boundaries. During the coding process, the paragraphs that appear more than 1 time were excluded from the data as inputs. Next, the large language model separated out the context-specific codes and assigned them to a category based on “context,” “sentiment,” “meaning,” and such similar features, as documented in the GPT-4.

## Results

### Summary of Data

Table 2 depicts the breach types that are common along with the type of incident shown. From this visualization, we can see that hacking or IT incidents are the types of breaches that have the highest impact in terms of the proportion of breaches. When considering the number of individuals affected, we see an even higher impact of hacking and IT-related incidents, which indicates hacking to be a continual issue in health care data privacy.

**Table 2:** Incidents and individuals by types of breaches.

Type of breach	Incidents (n=5158), n (%)	Individuals affected (n=382,533,378), n (%)
Hacking or IT incident	2465 (47.7)	316,771,372 (82.8)
Unauthorized access or disclosure	1268 (24.5)	24,074,273 (6.2)
Theft	999 (19.3)	26,876,573 (7)
Loss	220 (4.2)	9,449,646 (2.4)
Improper disposal	110 (2.1)	2,093,033 (0.5)
Other	84 (1.6)	1,188,272 (0.3)
Unknown	12 (0.2)	2,080,209 (0.5)

### Is HIPAA Implementation Alone Sufficient to Reduce Data Breaches?

We proposed a model with statewise fixed effects to analyze the role of the type of incidents and the location of the incident on the economic value of the affected party. We then used the number of affected individuals as the dependent variable indicating the economic effect of the breaches. Further, we used the health classification of states as either being HIPAA compliant or having local laws that are more stringent than HIPAA, which affect the number of individuals affected by the breach. The following equation specifies the model:

ln(Individuals affected) = α + β1-5 Type of breach indicator + β6-9 C.E. type + β10-60 State indicator + β61 StHIPAA

Table 3 contains 3 primary models, model 1, model 2, and model 3, which incrementally add variables (Multimedia Appendix 2). Model 1 is the baseline model. Model 2 adds additional covariates of health care provider, health care clearinghouse, and health plan. Model 3 includes the indicator stronger than HIPAA variable, which indicates whether the corresponding state-enabled rules are stronger than HIPAA.

**Table 3:** Regression models display the effect of the health care breach type on individuals affected^a^.

Dependent variable=ln(Individuals affected)	Model 1		Model 2		Model 3	
	β	*P* value	β	*P* value	β	*P* value
Hacking or IT incident	Included	—^b^	Included	—	Included	—
Improper disposal	–1.162^c^	<.001	–1.176^c^	<.001	–1.176^c^	<.001
Loss	–1.342^c^	<.001	–1.339^c^	<.001	–1.339^c^	<.001
Other	–1.014^c^	<.001	–1.085^c^	<.001	–1.085^c^	<.001
Theft	–1.168^c^	<.001	–1.218^c^	<.001	–1.218^c^	<.001
Unauthorized access or disclosure	–1.336^c^	<.001	–1.362^c^	<.001	–1.362^c^	<.001
Unknown	–1.249^d^	.02	–1.291^d^	.02	–1.291^d^	.02
Business associate	Included	—	Included	—	Included	—
Health plan	—	—	–0.382^c^	<.001	–0.382^c^	<.001
Health care clearing house	—	—	–0.393	.49	–0.393	.49
Health care provider	—	—	–0.411^c^	<.001	–0.411^c^	<.001
Stagewise fixed effect	Included	—	Included	—	Included	—
StHIPAA^e^	—	—		—	1.271^d^	.03
Constant	9.144^c^	<.001	8.743^c^	<.001	8.743^c^	<.001
Observations	5158	—	5140	—	5140	—
*R* ^2^	0.12	—	0.14	—	0.14	—

^a^Breach report is present in Multimedia Appendix 2.

^b^Not applicable.

^c^*P*<.001.

^d^*P*<.05.

^e^StHIPAA: stronger than Health Insurance Portability and Accountability Act.

From these results, we see that each of the separate types of vulnerability (ie, hacking or IT incident: β=9.144, improper disposal: β=–1.139, loss: β=–1.085, other: β=–1.218, theft: β=–1.362, and unauthorized access or disclosure: β=–1.291) significantly affect *ln(Individuals affected)*. From these, we see that the hacking or IT incident affects the number of individuals affected the highest, followed by loss of data, improper disposal, other types of hacking incidents, and theft of data. Overall, while different types of vulnerabilities affect and influence data uniquely, it is essential to observe that hacking or IT incident, which is the baseline category, has the highest effect on the number of individuals affected.

Similarly, the states that have laws that are significantly more stringent than HIPAA seem to be affected more by these different types of vulnerabilities (ie, stronger than HIPAA: β=1.271). Our results indicate that the higher the number of hacking and related incidents, the higher the chances for individuals being affected, and as a result, the most we need to protect against those types of attacks.

### Effect of the Omnibus Rule Amendment to HIPAA on Breaches Across US States

The 2013 Omnibus Rule offers an opportunity to examine how the changes implemented on January 17, 2013—captured by a variable where time=0 represents the pre hoc period and time=1 represents the post hoc period—affect the number of individuals impacted in both the treated states (ie, states with regulations stronger than HIPAA) and states adhering solely to HIPAA standards. Building upon models 1, 2, and 3, we use a difference-in-differences model to estimate how the “Omnibus” Rule affected *ln(Individuals affected)* in both the treatment and control groups. Our difference-in-differences econometric estimation is described in Table 4.

**Table 4:** The result of the difference-in-differences estimation.

	Model 1	*P* value^a^
Hacking or IT incident	Included	—^b^
Improper disposal	–1.170^c^	<.001
Loss	–1.330^c^	<.001
Other	–1.074^c^	<.001
Theft	–1.205^c^	<.001
Unauthorized access or disclosure	–1.361^c^	<.001
Unknown	–1.267^d^	.02
Business associate	Included	—
Health plan	–0.382^c^	<.001
Health care clearinghouse	–0.388	.50
Health care provider	–0.412^c^	<.001
Treated time (difference-in-differences effect)	0.0828	.50
Constant	8.741^c^	<.001
Observations	5140	—
*R* ^2^	0.14	—

^a^Two-tailed *t* test.

^b^Not applicable.

From our analysis, we observe that the overall difference-in-differences trend is not significant (coefficient=0.0828; *P*=.50). This demonstrates that despite laws being stronger than HIPAA in several states and the Omnibus Rule being issued, there are no guarantees that there will be a decrease overall, showing that despite additional guidelines and strengthening of protections, the number of individuals affected and breach trends continue to increase overall. Therefore, we can conclude that although HIPAA, state-specific stringent security rules, and Omnibus Rules provide guidelines, they are not sufficient on their own to prevent data breaches. Separate hospital-specific and breach-specific guidelines must also be followed.

### Qualitative Analysis Results

We performed a qualitative coding procedure on the description of each of the 4000+ data points using the artificial intelligence (AI)–assisted coding process. This method produces a substantial number of codes and likely more than what is perceived as useful data. To solve the problem, we used embeddings, a feature of language models for all the words or phrases that correspond to a point within multidimensional space. Using this technique, initially, similar words or phrases were clustered together. The system then repeatedly combined codes nearest to each other into a collection as clusters until an expected number of collections are achieved. For enhanced rigor, we developed and analyzed clusters of 9, 10, 11, 12, and 15 separate collections and manually examined the unique level 2 codes belonging to each collection for similarity and overlap. Two authors (HS and YX) separately analyzed the collections of codes and found that the 15 chosen categories had the lowest overlap and similarity among the choices above. We finalized 15 chosen categories, which are displayed in Table 5.

**Table 5:** Final set of categories with their weights from the coding analysis.

Type of breach	Weight (n=8517), n (%)
Data breach	2533 (30)
Security	1350 (16)
Privacy	1292 (15)
Protected health information	726 (9)
Communication	632 (7)
Regulatory compliance	355 (4)
Security breach	355 (4)
Risk management	305 (4)
Data privacy	234 (3)
Employee management	192 (2)
Access controls	177 (2)
IT	136 (2)
Policies	121 (1)
Procedures	71 (1)
Electronic communication	38 (0)

Table 5 shows the distribution of the generated codes in 15 categories representing the cluster of similar codes obtained from the analysis. From these results, we observed that the combination of data breach, security, and privacy accounts for more than 60% of the breach reports’ description, validating our original quantitative results.

**Figure 2:**
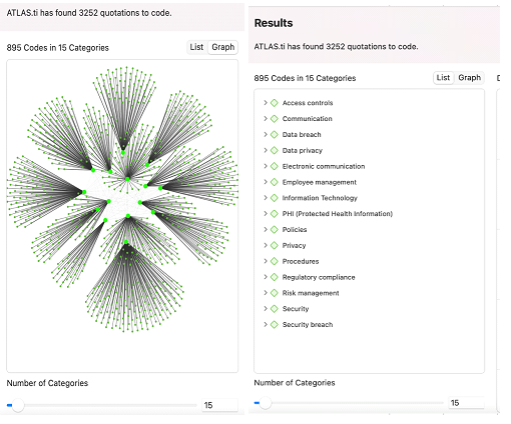
(A) Generated codes and clusters and (B) the list of codes and categories generated.

Figure 2 shows screen captures illustrating the process of category generation as embeddings in the ATLAS.ti user interface. Specifically, the larger neon green circles represent the categories, while the smaller green dots connected to them represent the relevant codes with similar characteristics.

We thus see 895 different codes created within the set of 15 categories. These categories correspond to the 15 underlying values based on the description of the breach report in the dataset: access controls, communication, data breach, data privacy, electronic communication, employee management, IT, PHI, policies, privacy, procedures, regulatory compliance, risk management, security, and security breach. By mapping these values onto their respective best practices as recommendations, we listed the definition of each core security-breach value as well as how organizations use these recommendations as exemplars in Multimedia Appendix 3 [24-85].

Based on the AI-generated summary from ATLAS.ti from the dataset in the health care industry, we find that the health care industry has faced numerous breaches of PHI, affecting a range of organizations such as hospitals, dental offices, insurance companies, and medical clinics. As a result, these breaches have led to unauthorized access, theft, and disclosure of sensitive personal and medical information through various means, including stolen laptops and physical documents, email errors, employee misconduct, and improper disposal of documents. In response to these incidents, investigations have been conducted by the Office for Civil Rights (OCR), and fines were imposed. Corrective actions have included enhancing data security measures, implementing encryption, conducting risk analyses, and providing breach notifications and credit monitoring to affected individuals. These breaches underscore the critical importance of safeguarding patient data, complying with HIPAA regulations, and continuously strengthening security protocols in the health care sector to prevent future incidents.

## Discussion

### Overview

Based on our quantitative and qualitative analyses of the breach data, we now discuss a further theoretical contribution of our paper that we call SecureSphere, a structured framework for health care security, based on the security values derived earlier.

### The SecureSphere Framework for Health Care Data System Security

**Figure 3:**
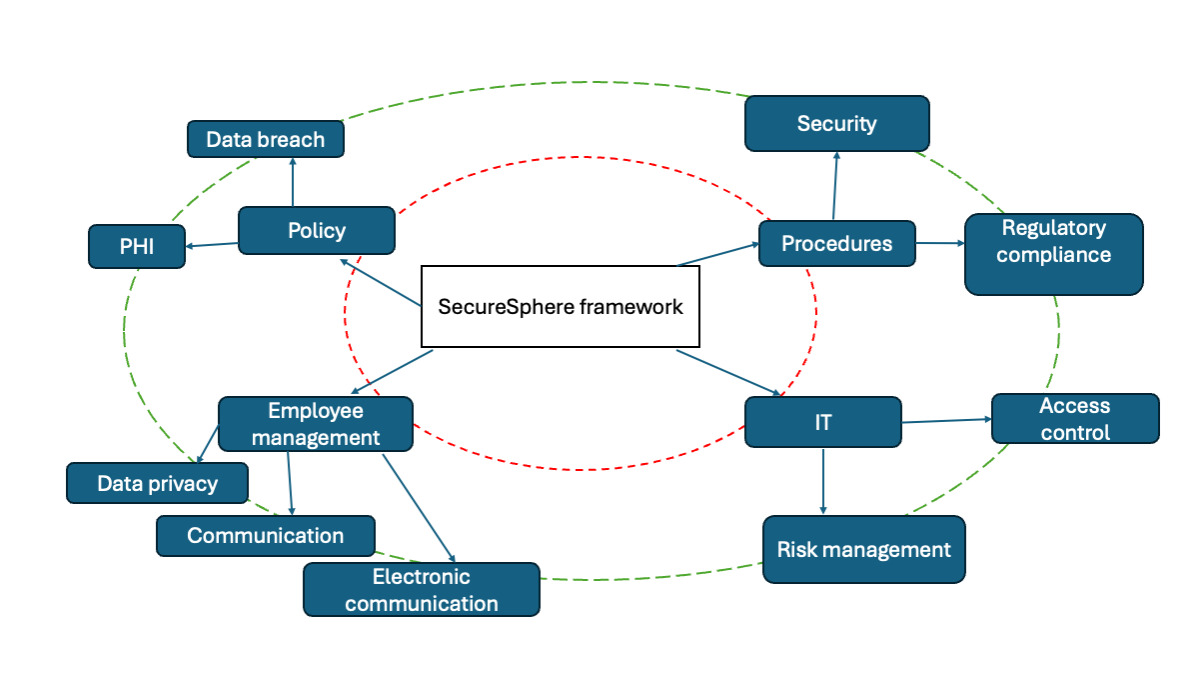
SecureSphere framework for Hospital Data Breach Protection. The inner circle represents the core values that guide and represent 80% of the breaches. The outer circle represents the peripheral values that form key business practices that influence the inner circle. PHI: protected health information.

The final step in our research is to classify the most important values that prevent large value data breaches overall and present the SecureSphere framework (Figure 3). The SecureSphere framework recommends a specific scope of action for the key security “values” and generic actions for preventing and sustaining data system security at hospitals and other health institutions that store, retrieve, and manage PHI.

We represent values in concentric circles. The innermost circle represents the core values that include employee management, policy, procedures, and IT. The outer circle represents peripheral values associated with each core value. Thus, the framework demonstrates how data breach and PHI influence policy, security, and regulatory compliance influence procedures within the hospital. Then, we have access control and risk management that influence IT procedures within a company. Finally, we have email communication, general communication, and data handling (privacy), which influence employee management.

While the core focal values are essential focus areas of health care companies, the outer values form the essential components within these focus areas. The SecureSphere framework identifies key security values and categorizes them into concentric circles to emphasize their influence on hospital data system security. The best practices by value category are summarized in Textboxes 1-3.

**Core focal values (inner circle).**

**Employee management**

- Training and awareness: Regular training programs to educate employees on security protocols and best practices.
- Access control: Implementing strict access controls to ensure that only authorized personnel can access sensitive data.
- Monitoring and auditing: Continuous monitoring of employee activities and regular audits to detect and prevent unauthorized actions.

**Policy**

- Comprehensive security policies: Developing clear and comprehensive security policies that outline acceptable use, data protection, and incident response.
- Policy enforcement: Ensuring strict enforcement of security policies through regular checks and penalties for noncompliance.
- Policy review and update: Regularly reviewing and updating policies to adapt to new threats and regulatory changes.

**Procedures**

- Standard operating procedures (SOPs): Creating detailed SOPs for handling data, responding to incidents, and managing IT infrastructure.
- Incident response procedures: Establishing clear procedures for responding to security incidents, including containment, investigation, and remediation.
- Regular audits: Conducting regular audits to ensure adherence to established procedures and identify areas for improvement.

**IT**

- IT infrastructure security: Implementing robust security measures for IT infrastructure, including firewalls, intrusion detection systems, and antivirus software.
- System hardening: Regularly updating and patching systems to protect against vulnerabilities.
- Incident response: Establishing an incident response team to quickly address and mitigate security breaches.

**Peripheral values (outer circle).**

**Data breach and protected health information (PHI)**

- Data encryption: Encrypting sensitive data to protect it from unauthorized access during transmission and storage.
- Data minimization: Collecting only the minimum necessary data to reduce the risk of breaches.
- PHI handling: Ensuring that PHI is handled in compliance with regulations such as HIPAA (Health Insurance Portability and Accountability Act).

**Regulatory compliance**

- Compliance monitoring: Regularly monitoring compliance with regulations such as HIPAA, general data protection regulation, and others.
- Compliance training: Providing ongoing training to employees on regulatory requirements and compliance practices.
- Reporting and documentation: Maintaining thorough documentation and reporting mechanisms to demonstrate compliance.

**Security**

- Layered security approach: Implementing multiple layers of security controls to protect data at various levels.
- Risk assessments: Conducting regular risk assessments to identify and mitigate potential security threats.
- Security best practices: Adopting industry best practices for securing networks, systems, and data.

**Procedures (influenced by policy, security, and regulatory compliance)**

- Regular procedure updates: Continuously updating procedures to reflect changes in policy, security requirements, and regulatory standards.
- Procedure training: Ensuring all employees are trained on the latest procedures and understand their roles in maintaining security.

**Additional influences.**

**Access control and risk management (influencing IT procedures)**

- Role-based access control (RBAC): Implementing RBAC to ensure users have access only to the information necessary for their role.
- Risk management strategies: Developing and implementing strategies to identify, assess, and mitigate risks.

**Email communication, general communication, and data handling (influencing employee management)**

- Secure email practices: Using encryption and secure email gateways to protect communication.
- Communication policies: Establishing clear policies for secure communication, both within and outside the organization.
- Data handling best practices: Implementing best practices for data handling, including secure storage, transmission, and disposal.

By following these best practices, hospitals can effectively enhance their data system security, minimize the risk of breaches, and ensure compliance with relevant regulations.

### Conclusions

This paper uses the HIPAA privacy and security rules and analyzes various types of breaches in the US health care system. Though HIPAA rules are stringent, they are not sufficient to prevent some of the basic types of vulnerabilities that influence and affect the major players overall. Our comprehensive analysis of the HIPAA privacy and security rules in conjunction with a detailed study of various types of breaches within the US health care system reveals a complex landscape. Despite the robust framework provided by HIPAA and subsequent amendments like the Omnibus Rule, breaches continue to occur with alarming frequency and impact. The persistence of these breaches underscores the necessity for continuous reassessment and enhancement of security protocols.

Our empirical analysis highlights that while regulatory frameworks form a critical backbone for data protection, they must be dynamically updated and rigorously enforced to adapt to evolving technological landscapes and sophisticated cyber threats. The addition of stringent state laws appears to correlate with better outcomes in some cases, suggesting that a multilayered approach to regulation may be beneficial.

Moreover, our findings advocate for the importance of fostering a culture of security within health care organizations that transcends compliance. Investing in cutting-edge technologies, training staff on a continuous basis, and taking a proactive stance on security are imperative.

The results of our detailed qualitative analysis of all text data provide support for 15 core values related to security breach management. These categories include access controls, communication, data breach, data privacy, electronic communication, employee management, IT, PHI, policies, privacy, procedures, regulatory compliance, risk management, security, and security breach. For each category, specific definitions, best practice recommendations, and exemplary organizations are identified. For instance, data breach involves unintentional disclosures, with Mayo Clinic cited for best practices like ongoing employee training and encryption measures. Regulatory compliance emphasizes aligning with laws, showcased by Deloitte’s ethics programs and Ernst & Young’s compliance management. IT highlights reducing uncertainty in organizational processes, with Apple noted for developing AI tools. This structured approach aims to enhance understanding and application of security measures in response to the frequent and varied breaches affecting health care entities.

Finally, it is evident that to truly safeguard patient data, collaboration across various stakeholders—including health care providers, patients, technology firms, and policy makers—is crucial. By integrating insights from this extensive analysis and categorizing values into the inner core and peripheral values, we present the SecureSphere framework for the health care industry, which can better protect itself against future breaches and ensure the integrity and privacy of sensitive health information. We highlight key issues with the framework that enables health care executives to make key decisions to enable better health care IT system security.

## Appendix

Multimedia Appendix 1 Details about human–artificial intelligence intercoder reliability with subsample of the dataset.

Multimedia Appendix 2 Breach report.

Multimedia Appendix 3 Values for information security practices with exemplars.

## Abbreviations

AI: artificial intelligence
EHR: electronic health record
HHS: Department of Health and Human Services
HIPAA: Health Insurance Portability and Accountability Act
PHI: protected health information
PHR: personal health record
