# Supplementary material for: Patient Health Record Protection Beyond the Health Insurance Portability and Accountability Act: Mixed Methods Study
Source: J Med Internet Res. 2024 Nov 6;26:e59674. doi: 10.2196/59674 (PMC11579621; doi:10.2196/59674)
Supplement: Multimedia Appendix 1 [file jmir_v26i1e59674_app1.docx]

Table S1.

| **Name** | **Kappa** | **Agreement** | **A And B (%)** | **Not A And Not B (%)** | **Disagreement** | **A And Not B (%)** | **B And Not A (%)** |
| --- | --- | --- | --- | --- | --- | --- | --- |
| Nodes\\Data breach | 0.95 | 98.19 | 24.55 | 73.64 | 1.82 | 0 | 1.82 |
| Nodes\\Data Privacy | 1 | 100 | 3.64 | 96.36 | 0 | 0 | 0 |
| Nodes\\Employee management | 1 | 100 | 9.09 | 90.91 | 0 | 0 | 0 |
| Nodes\\PHI | 0.93 | 98.64 | 10.91 | 87.73 | 1.36 | 1.36 | 0 |
| Nodes\\Privacy | 0.62 | 85.46 | 17.73 | 67.73 | 14.55 | 1.82 | 12.73 |
| Nodes\\Security | 0.94 | 98.18 | 17.27 | 80.91 | 1.82 | 0 | 1.82 |
| Nodes\\Security breach | 1 | 100 | 5.45 | 94.55 | 0 | 0 | 0 |
| Overall Unweighted Kappa | 1 | 100 | 3.64 | 96.36 | 0 | 0 | 0 |

In this qualitative analysis, the output of the Overall Unweighted Kappa was generated using NVIVO 14 with a sample size of n=50, serving to evaluate the intercoder reliability. Two distinct user profiles were utilized for the comparison between manual coding and ATLAS.TI AI coding. The first user profile, identified as yilxu with the initials YILIN, was coded manually based on 15 categories from ATLAS.TI coding. The second user profile is labeled as ATLASTI with the initials ATI which uses the ATLAS.TI 23 AI coding function with ChatGPT 3.5.

There are several potential weaknesses in the qualitative analysis that could be refined in future studies. One of the possible issues is that the ATLAS.TI AI coding does not specify the exact positions of the codes within the text which requires manual intervention to appropriately label the ATLAS.TI codes. Additionally, the codebook used for manual coding was developed based on categories generated from ATLAS.TI results target to establish common ground.

Figure S1.


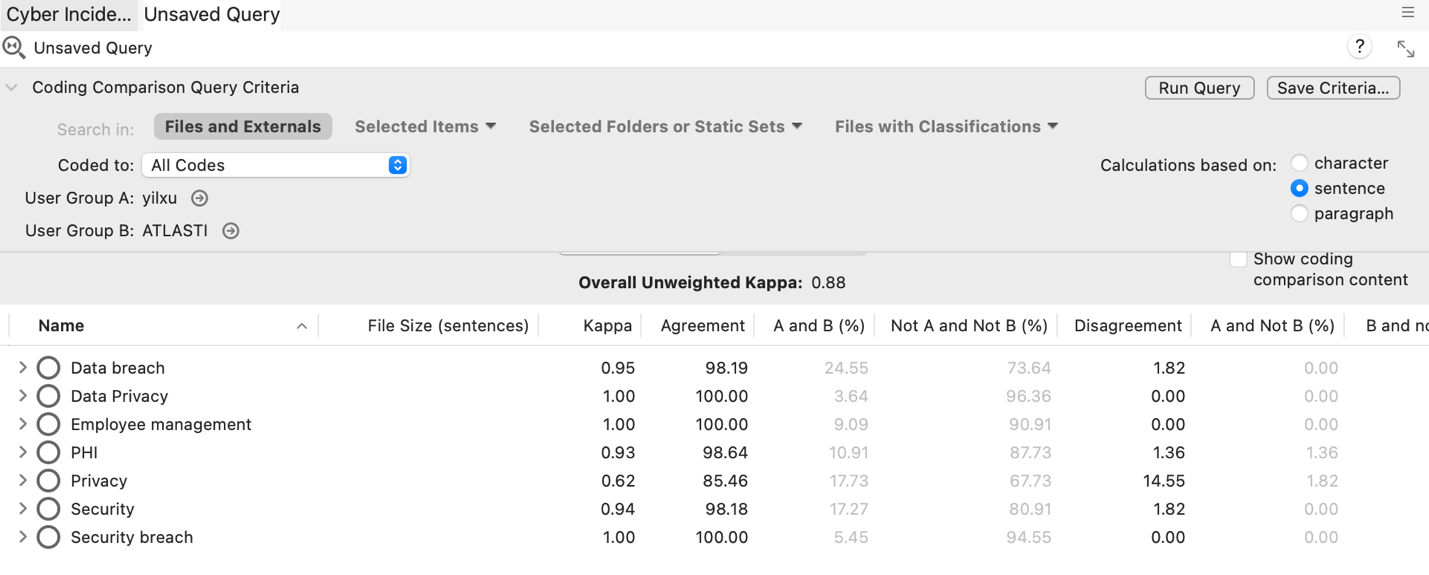


**Table S2**. Values for information security practices with exemplars.

| Security value | Definition | Best practice recommendation | Exemplars |
| --- | --- | --- | --- |
| Data Breach | Intentional or accidental disclosure of confidential information to individuals or entities who are not authorized to access it [24]. | - Ongoing Employee Training - Vendor Due Diligence - Encryption Measures | - Mayo Clinic [25] - Mayo Clinic [26] - Mayo Clinic [27] |
| Security | Perceived assurance of protection and stability depends on individual sentiments [28] | - Endpoint protection to access content - Design Zone to simplify security strategy and deployment - Security awareness training & phishing simulations | - Symantec [29] - Cisco [30] - Infosec institute [31] |
| Privacy | Fundamental right, crucial to freedom and well-being [32] | - Advance regulatory compliance for compliance Guidance - Improving transparency and empowering users - Improving privacy education for new users - Anonymization techniques by generalizing and adding noise to data | - United States Department of Health and Human Services [33] - Apple Inc [34] - Facebook [35] - Google [36] |
| Protected Health Information (PHI) | Health information that identifies an individual and is held or transmitted by a covered entity or its business associates [37] | - Encryption and transmitted information - Create risk assessments framework - Notice of privacy practice | - Epic Systems [38] - Cleveland Clinic [39] - Kaiser Permanente [40] |
| Communication | A self-sustaining process of information exchange within specific network designed for interactions [41] | - Ensure communication secure by State-of-the-art end-to-end encryption - Promote regular team meetings and status updates - Conduct online training for effective communication techniques | - Signal [42] - Atlassian [43] - LinkedIn Learning [44] |
| Regulatory compliance | Ensuring operations of organization align with applicable laws and regulations [45] | - Establish Ethics and compliance programs - Through Regulatory and Compliance Manager (EYRCM) digitally manage compliance within a shifting regulatory landscape - Provide routine internal audits, risk, and assessments | - Deloitte [46] - Ernst & Young [47] - KPMG [48] |
| Security breach | Confidentiality, integrity, and authentication leak from an organization’s network or computers [49] | - Ensure an incident response planning - Conduct post-breach analysis - Provide credit monitoring services as breach resolution | - IBM [50] - FireEye [51] - Experian [52] |
| Risk management | A defensive response addressing consumer and stakeholder expectations in organizational environment [53] | - ISO 31000 as international standard issued to guide. - Integrating risk management into corporate governance - Develop a risk communication plan for data breaches | - PECB [53] - Enterprise Risk Management Initiative [54] - PUSHKINPR [55] |
| Data privacy | Ensuring released data meets specific numerical or semantic non-identifiability criteria [56] | - Adopt approach based on principles to meet privacy commitment. - Conduct Privacy Impact Assessments(PIAs) - Ensure transparency in data processing and detail process as trust principle | - Microsoft [57] - Apple Inc [58] - Google Cloud [59] |
| Employee management | Implementation of human resource policies and practices to develop skills of employees to meet the needs of customers [60] | - Foster a positive and inclusive workplace culture - Provide training services - Implement fair performance management system with flexible process - provide small business guide to internal communication - Corporate culture priority work life balance of employee | - Google [61] - Siemens [62] - Adobe [63] - Salesforce [64] - Patagonia [65] |
| Access controls | Restricts use of information from users by authentication within a system [66] | - Apply multi-factor authentication(MFA) - Implement policy to control access to resources - Use role-based access control(RBAC) systems | - Microsoft [67] - Amazon Web Services [68] - Okta [69] |
| IT | Use tool for transforming inputs into outputs in organizational processes to reduce uncertainty [70] | - Develop emerging technology based on AI tools - Prioritize IT asset with zero trust - Invest in scalable cloud infrastructure | - Apple Inc [71] - Palo Alto Networks [72] - Amazon Web Services [73] |
| Policies | An organizational guideline to make decisions or the terms of an insurance contract [74] | - Develop fair and accessible policies - Regularly updated policy to guarantee relevant - Design policies as framework to inform customers | - Google [75] - IBM [76] - Salesforce [77] |
| Procedures | How to handle tasks and usually only apply to a single role [78] | - Using Enterprise class services to update system - Use product stewardship framework to continually improve and innovate procedures - Developer documents style allow procedures are documented and accessible | - IBM [79] - 3M [80] - Google [81] |
| Electronic communication | Direct method of information exchange from computer to computer [82] | - Foster effective internal and external digital Improve collaboration and efficacy in the workplace communication - Ensure Provide general data protection regulation (GDPR) to compliance with data protection laws - Implement Internet Relay Chat (IRC) channels | - Slack [83] - Microsoft [84] - Signal [85] |
