# Supplementary material for: Patient Health Record Protection Beyond the Health Insurance Portability and Accountability Act: Mixed Methods Study
Source: J Med Internet Res. 2024 Nov 6;26:e59674. doi: 10.2196/59674 (PMC11579621; doi:10.2196/59674)
Supplement: Multimedia Appendix 3 [file jmir_v26i1e59674_app3.docx]

Multimedia Appendix 3.

| Security value | Definition | Best practice recommendation | Exemplars |
| --- | --- | --- | --- |
| Data Breach | Intentional or accidental disclosure of confidential information to individuals or entities who are not authorized to access it [24]. | - Ongoing Employee Training - Vendor Due Diligence - Encryption Measures | - Mayo Clinic [25] - Mayo Clinic [26] - Mayo Clinic [27] |
| Security | Perceived assurance of protection and stability depends on individual sentiments [28] | - Endpoint protection to access content - Design Zone to simplify security strategy and deployment - Security awareness training & phishing simulations | - Symantec [29] - Cisco [30] - Infosec institute [31] |
| Privacy | Fundamental right, crucial to freedom and well-being [32] | - Advance regulatory compliance for compliance Guidance - Improving transparency and empowering users - Improving privacy education for new users - Anonymization techniques by generalizing and adding noise to data | - United States Department of Health and Human Services [33] - Apple Inc [34] - Facebook [35] - Google [36] |
| Protected Health Information (PHI) | Health information that identifies an individual and is held or transmitted by a covered entity or its business associates [37] | - Encryption and transmitted information - Create risk assessments framework - Notice of privacy practice | - Epic Systems [38] - Cleveland Clinic [39] - Kaiser Permanente [40] |
| Communication | A self-sustaining process of information exchange within specific network designed for interactions [41] | - Ensure communication secure by State-of-the-art end-to-end encryption - Promote regular team meetings and status updates - Conduct online training for effective communication techniques | - Signal [42] - Atlassian [43] - LinkedIn Learning [44] |
| Regulatory compliance | Ensuring operations of organization align with applicable laws and regulations [45] | - Establish Ethics and compliance programs - Through Regulatory and Compliance Manager (EYRCM) digitally manage compliance within a shifting regulatory landscape - Provide routine internal audits, risk, and assessments | - Deloitte [46] - Ernst & Young [47] - KPMG [48] |
| Security breach | Confidentiality, integrity, and authentication leak from an organization’s network or computers [49] | - Ensure an incident response planning - Conduct post-breach analysis - Provide credit monitoring services as breach resolution | - IBM [50] - FireEye [51] - Experian [52] |
| Risk management | A defensive response addressing consumer and stakeholder expectations in organizational environment [53] | - ISO 31000 as international standard issued to guide. - Integrating risk management into corporate governance - Develop a risk communication plan for data breaches | - PECB [53] - Enterprise Risk Management Initiative [54] - PUSHKINPR [55] |
| Data privacy | Ensuring released data meets specific numerical or semantic non-identifiability criteria [56] | - Adopt approach based on principles to meet privacy commitment. - Conduct Privacy Impact Assessments(PIAs) - Ensure transparency in data processing and detail process as trust principle | - Microsoft [57] - Apple Inc [58] - Google Cloud [59] |
| Employee management | Implementation of human resource policies and practices to develop skills of employees to meet the needs of customers [60] | - Foster a positive and inclusive workplace culture - Provide training services - Implement fair performance management system with flexible process - provide small business guide to internal communication - Corporate culture priority work life balance of employee | - Google [61] - Siemens [62] - Adobe [63] - Salesforce [64] - Patagonia [65] |
| Access controls | Restricts use of information from users by authentication within a system [66] | - Apply multi-factor authentication(MFA) - Implement policy to control access to resources - Use role-based access control(RBAC) systems | - Microsoft [67] - Amazon Web Services [68] - Okta [69] |
| IT | Use tool for transforming inputs into outputs in organizational processes to reduce uncertainty [70] | - Develop emerging technology based on AI tools - Prioritize IT asset with zero trust - Invest in scalable cloud infrastructure | - Apple Inc [71] - Palo Alto Networks [72] - Amazon Web Services [73] |
| Policies | An organizational guideline to make decisions or the terms of an insurance contract [74] | - Develop fair and accessible policies - Regularly updated policy to guarantee relevant - Design policies as framework to inform customers | - Google [75] - IBM [76] - Salesforce [77] |
| Procedures | How to handle tasks and usually only apply to a single role [78] | - Using Enterprise class services to update system - Use product stewardship framework to continually improve and innovate procedures - Developer documents style allow procedures are documented and accessible | - IBM [79] - 3M [80] - Google [81] |
| Electronic communication | Direct method of information exchange from computer to computer [82] | - Foster effective internal and external digital Improve collaboration and efficacy in the workplace communication - Ensure Provide general data protection regulation (GDPR) to compliance with data protection laws - Implement Internet Relay Chat (IRC) channels | - Slack [83] - Microsoft [84] - Signal [85] |

References:

24. Cheng L, Liu F, Yao DD. Enterprise data breach: causes, challenges, prevention, and future directions. WIREs Data Min Knowl Discov. 2017;7(5):e1211. [doi: 10.1002/widm.1211]

25. Avis E. Mayo Clinic's focus on staff needs, training and efficiency pays off. Health Facilities Management. 2022. URL: https://www.hfmmagazine.com/articles/4601-mayo-clinics-focus-on-staff-needs-training-and-efficiency-pays-off [accessed 2024-10-10]

26. Loughlin S. In contracts with device vendors, Mayo Clinic emphasizes security. Biomed Instrum Technol. 2016;50(1):53-55. [doi: 10.2345/0899-8205-50.1.53] [Medline: 26829140]

27. Privacy policy. Mayo Clinic. 2024. URL: https://www.mayoclinic.org/about-this-site/privacy-policy [accessed 2024-10-10]

28. Rothchild E. What is security? Daedalus. 1995;124(3):53-98.

29. We stop threats hiding in plain sight. Symantec. 2024. URL: https://sep.securitycloud.symantec.com/v2/landing [accessed 2024-10-10]

30. Design zone for security. Cisco. 2024. URL: https://www.cisco.com/c/en/us/solutions/enterprise/design-zone-security/ index.html#:~:text=The%20Cisco%20Design%20Zone%20for,defense%2C%20and%20other%20security%20architectures [accessed 2024-10-10]

31. Infosec. 2024. URL: https://www.infosecinstitute.com/iq/welcome/ [accessed 2024-10-10]

32. Solove DJ. Understanding Privacy. United States. Harvard University Press; 2010:272.

33. Compliance guidance. US Department of Health and Human Services Office of Inspector General. 2023. URL: https://oig. hhs.gov/compliance/compliance-guidance/ [accessed 2024-10-10]

34. Data privacy day at Apple: improving transparency and empowering users. Apple Newsroom. 2021. URL: https://www. apple.com/bh/newsroom/2021/01/data-privacy-day-at-apple-improving-transparency-and-empowering-users/ [accessed 2024-10-10]

35. Martinez J. Facebook unveils privacy education feature for new users. The Hill. 2012. URL: https://thehill.com/policy/ technology/133497-facebook-unveils-privacy-education-feature-for-new-users/ [accessed 2024-10-10]

36. How Google anonymizes data. Google. URL: https://policies.google.com/technologies/anonymization?hl=en-US[accessed 2024-10-10]

37. Isola S, Al Khalili Y. Protected Health Information. Treasure Island, FL. StatPearls Publishing; 2024.

38. Security and privacy policies. Epic. 2023. URL: https://www.epic.com/privacypolicies/ [accessed 2024-10-10]

39. McGee MK. Cleveland Clinic: assessing risks. ISMG Network. 2013. URL: https://www.healthcareinfosecurity.com/ interviews/cleveland-clinic-assessing-risks-i-1820 [accessed 2024-10-10]

40. Notice of privacy practices. US Department of Health and Human Services. 2023. URL: https://www.hhs.gov/hipaa/ for-individuals/notice-privacy-practices/index.html [accessed 2024-10-10]

41. Luhmann N. What is communication? Commun Theory. 1992;2(3):251-259. [FREE Full text] [doi: 10.1111/j.1468-2885.1992.tb00042.x]

42. Share without insecurity. Signal. URL: https://signal.org/ #:~:text=State%2Dof%2Dthe%2Dart,%2C%20every%20call%2C%20every%20time [accessed 2024-10-10]

43. How to give your team meetings a status update. Atlassian. 2016. URL: https://www.atlassian.com/blog/teamwork/ give-team-meetings-status-update [accessed 2024-10-10]

44. Learning more effective communication techniques for your communication style. LinkedIn Learning Blog. 2023. URL: https://www.linkedin.com/business/learning/blog/effective-communication-techniques-for-your-communication-style [accessed 2024-10-10]

45. Kharbili ME, Ma Q, Kelsen P, Pulvermueller E. CoReL: policy-based and model-driven regulatory compliance management. IEEE; 2011. Presented at: 2011 IEEE 15th International Enterprise Distributed Object Computing Conference; August 29-September 2, 2011:247-256; Helsinki, Finland. [doi: 10.1109/edoc.2011.23]

46. Ethics and compliance programs. Deloitte. URL: https://www2.deloitte.com/br/en/pages/risk/solutions/ programas-de-etica-compliance.html [accessed 2024-10-10]

47. EY regulatory compliance manager. EY. 2024. URL: https://www.ey.com/en_gl/services/consulting/ regulatory-compliance-manager [accessed 2024-10-10]

48. Internal audit, risk and compliance services. KPMG. 2024. URL: https://kpmg.com/dp/en/home/services/advisory/ risk-consulting/internal-audit-risk-compliance-services. html#:~:text=KPMG's%20Internal%20audit%2C%20Risk%20%26%20compliance,supply%20chain%20to%20augment%20and [accessed 2024-10-10]

49. Hovav A, Andoh-Baidoo F, Dhillion G. Classification of security breaches and their impact on the market value of firms. 2007. Presented at: Proceedings of the 6th Annual Security Conference; 2007; Las Vegas, NV, United States.

50. Cyber incident response planning. IBM. 2021. URL: https://www.ibm.com/docs/en/ ftmswsfz300?topic=program-cyber-incident-response-planning [accessed 2024-10-10]

51. Maude MMJ. A post breach analysis: Okta support unit. BeyondTrust. 2024. URL: https://www.beyondtrust.com/webinars/ a-post-breach-analysis-okta-support-unit [accessed 2024-10-10]

52. Global data breach resolution. Experian. 2023. URL: https://www.experian.com/data-breach/solutions/ global-data-breach-resolution [accessed 2024-10-10]

53. Power M. The risk management of everything. J Risk Fin. 2004;5(3):58-65. [doi: 10.1108/eb023001] 54. Enterprise Risk Management Initiative. NC State University. 2024. URL: https://erm.ncsu.edu/ [accessed 2024-10-10]

55. Neelis M. A crisis communications plan for data breaches. PUSHKINPR Authentic Communication. URL: https://www. pushkinpr.com/blog/crisis-communications-plan-data-breaches/ [accessed 2024-10-10]

56. Vimercati SDCD, Foresti S, Livraga G, Samarati P. Data privacy: definitions and techniques. Int J Unc Fuzz Knowl Based Syst. 2012;20(06):793-817. [doi: 10.1142/s0218488512400247]

57. Privacy and data management overview. Microsoft. 2024. URL: https://learn.microsoft.com/en-us/compliance/assurance/ assurance-privacy [accessed 2024-10-10]

58. Privacy governance. Apple. URL: https://www.apple.com/legal/privacy/en-ww/governance/ [accessed 2024-10-10]

59. Creating trust through transparency. Google Privacy. URL: https://cloud.google.com/transparency [accessed 2024-10-10]

60. Blount Y. Employee management and service provision: a conceptual framework. Inf Technol People. 2011;24(2):134-157. [doi: 10.1108/09593841111137331]

61. Who we are shapes the work we do. Google Belonging. URL: https://about.google/belonging/at-work/ [accessed 2024-10-10]

62. Training services—life is for learning, let's turn it into success. Siemens. URL: https://www.siemens.com/global/en/products/ services/digital-enterprise-services/training-services.html [accessed 2024-10-10]

63. HR lessons from the Adobe performance management overhaul. Performyard. 2023. URL: https://www.performyard.com/ articles/adobe-performance-management [accessed 2024-10-10]

64. The small business guide to internal communication. Salesforce. 2024. URL: https://www.salesforce.com/resources/articles/ small-business-guide-internal-communication/ [accessed 2024-10-10]

65. Bhayani T. Crafting culture: insights from the Patagonia employee handbook for your business. AirMason. 2024. URL: https://blog.airmason.com/patagonia-employee-handbook-pdf/ #:~:text=Patagonia%20champions%20employee%20empowerment%20and,special%20benefits%20for%20breastfeeding%20mothers [accessed 2024-10-10]

66. Sandhu RS, Samarati P. Access control: principle and practice. IEEE Commun Mag. 1994;32(9):40-48. [doi: 10.1109/35.312842]

67. Microsoft Entra multifactor authentication (MFA). Microsoft. URL: https://www.microsoft.com/en-us/security/business/ identity-access/microsoft-entra-mfa-multi-factor-authentication [accessed 2024-10-10]

68. Control access to AWS resources using policies. Amazon Web Services. URL: https://docs.aws.amazon.com/IAM/latest/ UserGuide/access_controlling.html [accessed 2024-10-10]

69. What is role-based access control (RBAC)? Okta. 2024. URL: https://www.okta.com/identity-101/ what-is-role-based-access-control-rbac/ #:~:text=Role%2Dbased%20access%20control%20(RBAC)%20systems%20assign%20access%20and,different%20roles%20have%20different%20rights [accessed 2024-10-10]

70. Dewett T, Jones GR. The role of information technology in the organization: a review, model, and assessment. J Manage. 2001;27(3):313-346. [doi: 10.1016/S0149-2063(01)00094-0]

71. Introducing Apple's on-device and server foundation models. Apple Machine Learning Research. 2024. URL: https:/ /machinelearning.apple.com/research/introducing-apple-foundation-models [accessed 2024-10-10]

72. Best practices implementing zero trust with Palo Alto Networks. paloaltoTECHDOCS. 2024. URL: https://docs. paloaltonetworks.com/best-practices/zero-trust-best-practices/zero-trust-best-practices/the-five-step-methodology/ step-1-asset-discovery-and-prioritization [accessed 2024-10-10]

73. AWS plans to invest 2.26 trillion yen into its Japanese cloud infrastructure by 2027. Amazon Web Services. 2024. URL: https://press.aboutamazon.com/aws/2024/1/ aws-plans-to-invest-2-26-trillion-yen-into-its-japanese-cloud-infrastructure-by-2027 [accessed 2024-10-10]

74. von Solms R, von Solms B. From policies to culture. Comput Secur. 2004;23(4):275-279. [FREE Full text] [doi: 10.1016/j.cose.2004.01.013]

75. Providing a safe and trusted experience for everyone. GooglePlay. URL: https://play.google/developer-content-policy/ [accessed 2024-10-10]

76. Modifying a policy. IBM. 2023. URL: https://www.ibm.com/docs/en/data-risk-manager/2.0.6?topic=policies-modifying-policy [accessed 2024-10-10]

77. Fenton E. Salesforce subprocessors: understanding key vendors and their roles. visualping; 2024. URL: https://visualping. io/blog/salesforce-subprocessors [accessed 2024-10-10]

78. Bandor MS. Process and procedure definition: a primer. Software Engineering Institute. 2007. URL: https://insights. sei.cmu.edu/documents/3263/2007_017_001_23937.pdf [accessed 2024-10-10]

79. Updating system software by using Enterprise Class services. IBM. 2024. URL: https://www.ibm.com/docs/en/ flashsystem-9x00/8.4.x?topic=uss-updating-system-software-by-using-enterprise-class-services [accessed 2024-10-10]

80. Our global impact—sustainability/ESG. 3M. 2024. URL: https://www.3m.com/3M/en_US/sustainability-us/governance/ innovation-management/ [accessed 2024-10-10]

81. Procedures. Google developer documentation style guide. 2024. URL: https://developers.google.com/style/procedures [accessed 2024-10-10]

82. van der Kam WJ, Moorman PW, Koppejan-Mulder MJ. Effects of electronic communication in general practice. Int J Med Inform. 2000;60(1):59-70. [doi: 10.1016/s1386-5056(00)00096-4] [Medline: 10974641]

83. Slack communication: enhancing collaboration and efficiency in the workplace. Cerkl. 2024. URL: https://cerkl.com/blog/ slack-communication/ [accessed 2024-10-10]

84. Safeguard individual privacy with cloud services from Microsoft. Microsoft. URL: https://www.microsoft.com/en-us/ trust-center/privacy/gdpr-overview#GDPR [accessed 2024-10-10]

85. The best ways to secure communication channels in the enterprise environment. Cybersecurity Insiders. URL: https://www. cybersecurity-insiders.com/the-best-ways-to-secure-communication-channels-in-the-enterprise-environment/ [accessed 2024-10-10]
